# Ethical leadership behaviors of senior managers perceived by the junior managers working in public hospitals in Hail, Saudi Arabia

**DOI:** 10.12669/pjms.40.5.8996

**Published:** 2024

**Authors:** Badr K. Aldhmadi, Rakesh Kumar, Bilesha Perera, Mohammad A. Algarni, Sage Mesias Raguindin, Ammar A. Attar, Guidoume Ahmed

**Affiliations:** 1 Dr. Badr K. Aldhmadi, Ph.D. Department of Health Management, College of Public Health and Health Informatics, University of Ha’il, Ha’il, Saudi Arabia; 2 Dr. Rakesh Kumar, Ph.D. Department of Health Management, College of Public Health and Health Informatics, University of Ha’il, Ha’il, Saudi Arabia; 3 Dr. Bilesha Perera, MSc., Ph.D Faculty of Medicine, University of Ruhuna, Galle, Sri Lanka; 4 Dr. Mohammad A. Algarni, Ph.D. Faculty of Economic and Administration, King Abdulaziz University, Jeddah, Saudi Arabia; 5 Dr. Sage Mesias Raguindin, RN. MN. PhD. College of Nursing, University of Ha’il, Ha’il, Saudi Arabia; 6 Dr. Ammar A. Attar, Ph.D. Department of Laboratory Medicine, Faculty of Applied Medical Sciences, Umm Al-Qura University, Makkah, Saudi Arabia; 7 Dr. Guidoume Ahmed, Ph.D. Department of Psychology and Sciences of Education, University of Mostaganem, Mostaganem, Algeria

**Keywords:** Ethical leadership, Health care managers, Perceptions, Saudi Arabia

## Abstract

**Objective:**

To examine junior managers experiences of ethical leadership behaviors exhibited by their senior managers.

**Methods:**

In this cross-sectional study, 263 junior health care managers working in public hospitals in Hail, Saudi Arabia were surveyed using a self-administered questionnaire between 20 November, 2022 and 15 February, 2023. Descriptive statistics and regression analysis were employed in the analysis. Statistical Package for Social Sciences (SPSS) version 25 (IBM, Armonk, NY, USA) was used to conduct statistical analyses.

**Results:**

The sample consisted of 118 men (44.9%) and the majority (66.6%) of the respondents were below the age of 36 years. In case of working environment, nearly 84% of the participants were satisfied with the relationships that they have had with their supervisors. Regression analysis indicate that women were more likely than men to experience healthy ethical leadership behaviors of their seniors (β = -0.163, p < 0.05). Ethical leadership behaviors of senior health care managers would not influence by the age or work experience of their juniors.

**Conclusion:**

Ethical leadership behavior of senior health care managers was satisfactory. Longitudinal research is needed to investigate how cultural and environmental factors affect the ethical leadership behavior of healthcare managers in Saudi Arabia.

## INTRODUCTION

Due to changing technology and emerging demands from the stakeholders, healthcare workforce is charged with more duties and responsibilities. Sincere, truthful and ethical conduct of employees is an important influence in shaping the organizational environment healthy, and to have integrated approach for better outcomes.^1^-^4^ In the past, leaders were considered extraordinary humans possessing extraordinary powers to motivate workers and keep them committed to their work and organizational improvements.^5^ However, in the last few decades, leadership qualities included achieving quality outcomes with a healthy healthcare workforce.^2^,^6^ Acting ethically and responsibly can lead to protecting corporate interests more than the self-interest of its members. Historically, leadership responsibilities and qualities were mainly described using situational and behavioral theories, in which leadership is defined based on situations and behaviors.^7^-^10^ Brown et al. (2006) proposed that ethical leadership is a distinct style and defined ethical leadership as “the demonstration of normatively appropriate conduct through personal actions and interpersonal relationships and the promotion of such behavior to followers through communication, reinforcement, and decision making”.^7^ While fulfilling the responsibilities in a healthcare setting, the healthcare leaders have to interact with many stakeholders and decision-making process without conflicting with ethical standards.^9^-^11^

It is observed that healthcare professionals do not pay adequate attention to unethical behaviors of their executives for many reasons including economic pressures, top leaders striving to achieve the set goals, greediness, and ignorance.^5^,^8^,^12^-^14^ A lack of concern about ethical behavior among healthcare leaders and their followers is a significant obstacle to quality healthcare delivery.^8^,^15^ There is a shortage of research data pertaining to ethical leadership behavior of healthcare managers in Saudi Arabia. The main objective of this study was to assess how junior managers perceive ethical leadership behavior of senior managers in hospital settings in Hail, Saudi Arabia.

## METHODS

The target population of the study was junior healthcare managers working in public hospitals in Hail, Saudi Arabia. A cross-sectional survey was conducted using a sample of junior health care managers attached to a public hospital. An anonymous questionnaire was used to collect data from respondents between 20 November, 2022 and 15 February, 2023.

### Ethical considerations:

Before commencing this study, ethical approval was obtained from University of Ha’il Research Ethics Committee (Number: H-2020-196) and Ministry of Health (IRB Registration Number: H-08-L-074) in Saudi Arabia.

### Informed consent:

Informed consent was obtained from all subjects involved in the study.

### Inclusion and exclusion criteria:

Those who had given informed consent were invited to participate in the study. Participants who were not willing or having any health issue at the time of the survey were excluded.

### Study Instruments and Variables:

The study instrument was a self-administered questionnaire. The survey questionnaire included demographic characteristics and 15 items Ethical Leadership Questionnaire (ELQ).^7^,^13^ The ELQ assessed participants’ experience with their immediate supervisor. The responses were recorded on a 6-point Likert scale (1 = strongly disagree to 6 = strongly agree). Items such as “My boss shows a strong concern for ethical and moral values” were included in the ELQ. The total score of this tool ranged between 15 and 90, where a higher score indicates a higher quality of ethical leadership. The English version of the ELQ was translated into Arabic. Both English and Arabic versions were included in the survey Form to ensure that the participants understood the questions clearly. A pilot test of the comprehensibility and understandability of the questionnaire was performed with five junior healthcare management staff members attached to another hospital in the area. The Cronbach’s alpha of the 15 items ELQ was 0.812. The ELQ is a reliable and valid scale that has been widely used in conducting surveys in many organizations.

### Sampling Procedure and Data Collection:

All junior healthcare managers in the selected public hospital were invited for the survey. Since there were no previous studies on ethical leadership in the area of healthcare management, the minimum sample size needed was calculated assuming that 80% of the respondents had good ethical leadership qualities and using a 95% confidence level with a margin of error of 5%. Thus, the minimum sample size required was 246.

### Data analysis:

Statistical Package for Social Sciences (SPSS) version 25 (IBM, Armonk, NY, USA) was used to conduct statistical analyses. The level of statistical significance was set as p < 0.05. Frequencies and percentages were computed to examine categorical variables and means and standard deviations were computed to examine continuous variables. Hierarchical linear regression was performed to predict the ethical leadership behavior of seniors perceived by junior managers working in health care setting based on the demographic profile of the junior managers.

## RESULTS

It was seen that there is slightly higher percentage of females in the sample, reflecting gender inequivalence in the population of healthcare managers in Hail region (Table-I). Nearly four fifth of the participants were in the middle age group. The majority were married and having a bachelor or a higher degree. About 68% of the participants were having 10 or less than 10 years of work experience.

**Table-I:** Characteristics of sample subjects (n= 263).

Characteristic	Number	Percentage
***Gender***		
Male	118	44.9
Female	145	55.1
***Age***		
Not given	8	3.0
20-25	17	6.5
26-30	83	31.5
31-35	75	28.5
36-40	48	18.3
>40	32	12.2
***Marital status***		
Not given	4	1.5
Single	79	30.0
Married	180	68.5
***Level of Education***		
High school	5	1.9
Diploma	53	20.2
Bachelor	155	58.9
Master	30	11.4
Doctorate	20	7.6
***Years of Experience***		
Not given	6	2.3
1-4 Years	64	24.3
5-10 Years	115	43.7
5-10 >10 Years	78	29.7

It seems that the majority of the respondents were satisfied with their physical health and having good relationship with their peers and their supervisors. Job stresses a major component related to good working condition and job satisfaction were experience by nearly 42.3% of the participants. Nearly a half of the participants were not satisfied with their salary. Table-II

**Table-II:** Views about working environment (n=263).

Statement	Strongly disagree n (%)	Moderately disagree n (%)	Slightly disagree n (%)	Slightly agree n (%)	Moderately agree e n (%)	Strongly agree n (%)
My Job stress is high.	24 (9.1)	29 (11.0)	28 (10.6)	71 (27.0)	58 (22.1)	53 (20.2)
My peer relationships are excellent.	3 (1.1)	2 (0.8)	8 (3.0)	22 (8.4)	55 (20.9)	173 (65.8)
My relationships with my superiors are excellent.	4 (1.5)	4 (1.5)	5 (1.9)	28 (10.6)	54 (20.6)	168 (63.9)
My salary and other benefits are satisfying.	26 (9.9)	27 (10.3)	32 (12.2)	48 (18.3)	75 (28.5)	55 (20.8)
My Physical Health is excellent.	4 (1.5)	6 (2.4)	20 (7.7)	37 (14.2)	60 (22.8)	136 (51.4)

It seems that ethical leadership behavior seen in this sample is satisfactory (Table-III). About three forth of the participants reported that their immediate supervisor has having good ethical leadership behavior. Consistency, fair evaluation and motivational efforts seen in their leaders were somewhat exceptional.

**Table-III:** Responses for the Ethical Leadership Questionnaire (n=263*).

Statement	Strongly disagree n (%)	Moderately disagree n (%)	Slightly disagree n (%)	Slightly agree n (%)	Moderately agree e n (%)	Strongly agree n (%)
My boss shows a strong concern for ethical and moral values	2 (0.8)	4 (1.5)	8 (3.0)	24 (9.1)	56 (21.3)	169 (64.3)
My boss communicates clear ethical Standards for members	2 (0.8)	4 (1.5)	10 (3.8)	23 (8.7)	55 (20.9)	169 (64.3)
My boss sets an example of ethical behavior in his/her decisions and actions	5 (1.9)	5 (1.9)	11 (4.2)	26 (9.9)	51 (19.4)	165 (62.7)
My boss is honest and can be trusted to tell the truth.	5 (1.9)	8 (3.0)	9 (3.4)	33 (12.5)	39 (14.8)	169 (64.3)
My boss keeps his/her actions consistent with his/her stated values (“walks the talk”)	9 (3.4)	4 (1.5)	11 (4.2)	39 (14.8)	45 (17.1)	154 (58.6)
My boss is fair and unbiased when assigning tasks to members.	10 (3.8)	5 (1.9)	12 (4.6)	33 (12.5)	44 (16.7)	159 (60.5)
My boss is fair and unbiased when assigning tasks to members.	7 (2.7)	3 (1.1)	13 (4.9)	30 (11.4)	48 (18.3)	161 (61.2)
My boss insists on doing what is fair and ethical even when it is not easy	11 (4.2)	4 (1.5)	10 (3.8)	33 (12.5)	49 (18.6)	156 (59.3)
My boss acknowledges mistakes and takes responsibility for them	6 (2.3)	11 (4.2)	12 (4.6)	37 (14.1)	34 (12.9)	162 (61.6)
My boss regards honesty and integrity as important personal values	7 (2.7)	4 (1.5)	9 (3.4)	26 (9.9)	34 (12.9)	183 (69.6)
My boss sets an example of dedication and self-sacrifice for the organization	7 (2.7)	5 (1.9)	12 (4.6)	29 (11.0)	42 (16.0)	168 (63.9)
My boss opposes the use of unethical practices to increase performance.	8 (3.0)	5 (1.9)	16 (6.1)	23 (8.7)	37 (14.1)	174 (66.2)
My boss is fair and objective when evaluating member performance and providing rewards	5 (1.9)	5 (1.9)	9 (3.4)	31 (11.8)	47 (17.9)	165 (62.7)
My boss puts the needs of others above his/her own self-Interest	13 (4.9)	5 (1.9)	9 (3.4)	33 (12.5)	55 (20.9)	148 (56.3)
My boss holds members accountable for using ethical practices in their work.	7 (2.7)	12 (4.6)	13 (4.9)	27 (10.3)	40 (15.2)	164 (62.4)

* In some statement one or two subjects had not responded.

The mean score of the ELQ for the entire sample was 79.1 (*SD* = 15.5). Among men the figure was 81.4 (*SD* = 12.8) and among women the corresponding figure was 77.1 (*SD* = 17.2), A significant gender difference of the ELQ score was observed (*p* < 0.05). Regression analysis indicate that neither the age nor the number of years of working experience were significant predictors of ethical leadership behavior. Regression analysis indicate that female participants were more likely than male participants to experience healthy ethical leadership behavior (Table-IV).

**Table-IV:** Hierarchical linear regression for the outcome variable total Score of ELQ.

		B	Std. Error	β	t	Sig
Step1	Constant	79.157	1.719		46.042	.000
	Experience	-.016	.158	-.006	-.098	.922
Step 2	Constant	80.240	3.001		26.739	.000
	Experience	.060	.232	.024	.256	.798
	Age	-.584	1.326	-.042	-.441	.660
Step 3	Constant	89.967	4.915		18.303	.000
	Experience	.084	.230	.034	.367	.714
	Age	-1.264	1.340	-.090	-.943	.347
	Gender	-5.138	2.069	-.163	-2.483	.014*

Dependent Variable: Ethical Leadership,

* p < 0.05.

## DISCUSSION

The findings of this study indicated that leaders’ (senior healthcare managers) ethical leadership behavior is satisfactory as perceived by the junior health care managers and it was consistent with the results of other researchers.^15^-^17^ Although, demographic and socio-cultural factors influence ethical leadership behavior of healthcare work force,^8^,^15^,^18^ in this study only gender was found to be a factor associated with ethical leadership behavior of management staff working in healthcare settings in Hail, Saudi Arabia. Most of the previous studies have been conducted in government offices, firms, schools and universities^15^,^19^-^22^ and therefore the results of the current study, which was conducted in a public hospital, is important to understand leadership behaviors of health care managers in Saudi Arabia

Healthcare systems in the world are changing rapidly, giving more attention to create low-cost, more effective healthcare systems. Healthcare management play a vital role in shaping health care deliveries more attractive and user-friendly. Since female management staff is more likely than their male counterparts to experience healthy and ethically sound leadership behavior and practices of their seniors, when improving healthcare management systems by addressing ethical leadership qualities of junior and senior management officers in hospitals gender should be considered as an important factor, and male healthcare managers should be educated and motivated to develop ethical leadership behaviors. In parallel with our results, studies have demonstrated that there is a perceived gender differences exist in ethics^23^ and which is usually in favor of females.^24^,^25^ Age nor the work experience has any association with ethical leadership behaviors of senior health care managers in Hail, Saudi Arabia. Adrian (2017) explored that age is not having any relationship with ethical behavior.^26^ Borkowski et al. and Ugras et al. (1992) found no correlation between work experience and ethical conduct.^27^ Personality, working environment and facilities available are other important factors that needs to be considered when changing attitudes of healthcare mangers towards to have ethically sound workforce.^4^,^11^,^22^

### Limitations:

It is not possible to generalize these results because we have used only public healthcare setting for this study. There may be a response bias as evaluating seniors may be a sensitive issue for the respondents. This is a cross sectional study and therefore cannot make any cause-effect relationships.

## CONCLUSION

Our results showed that ethical leadership behavior of senior healthcare managers in Hail, Saudi Arabia were satisfactory according to the junior health care managers and therefore, attention should be paid by health authorities to maintain such work atmosphere. Results also indicates that female participants more than their male counterparts strongly perceived that their senior healthcare managers have high ethical conduct, thus educational interventions are needed to enhance ethical leadership qualities of male healthcare managers. Ethical leadership behavior should be viewed as an important issue in achieving quality healthcare in Saudi Arabia. Further, longitudinal research is recommended to investigate how cultural and environmental factors affect the ethical leadership behavior of healthcare managers in Saudi Arabia.
